# von Willebrand Factor (VWF) Inhibitors in Two Brothers with von Willebrand Disease: A Case Report

**DOI:** 10.1055/a-2606-9625

**Published:** 2025-06-06

**Authors:** Claudia Djambas Khayat, Anna Pavlova, Sylvia Werner, Sigurd Knaub, Robert F. Sidonio

**Affiliations:** 1 Hotel Dieu de France Hospital, Saint Joseph University, Beirut, Lebanon; 2 Institute of Experimental Haematology and Transfusion Medicine, University Clinic Bonn, Bonn, Germany; 3 Octapharma USA, Paramus, New Jersey, United States; 4 Octapharma AG, Lachen, Switzerland; 5 Department of Pediatrics, Emory University School of Medicine, Atlanta, Georgia, United States

**Keywords:** von Willebrand disease, plasma-derived von Willebrand factor, prophylaxis, von Willebrand factor inhibitors

## Abstract

The development of inhibitors to von Willebrand factor (VWF) is a rare but potentially serious complication of VWF replacement therapy in patients with von Willebrand disease (VWD). Patients who develop VWF inhibitors may become unresponsive and/or may develop severe anaphylactic reactions to VWF concentrates. Data on inhibitor development and management in VWD remain limited, and better understanding of inhibitor development is an important goal in VWD management. The WIL-31 study demonstrated the efficacy and safety of prophylaxis with wilate, a plasma-derived VWF/factor VIII (pdVWF/FVIII) concentrate, in children and adults with VWD of all types. The annualized bleeding rate (ABR) was reduced by 84% with wilate prophylaxis compared with on-demand treatment, and prophylaxis was well tolerated. No inhibitors developed during the WIL-31 study. Here, we report two brothers with type 3 VWD who at the 6-month visit were found to have VWF inhibitors, which on further investigation were found to have already been present before the study. Despite the presence of inhibitors, neither patient showed any clinical symptoms, and prophylaxis with wilate led to a ≥85% reduction in ABR in both boys compared with on-demand treatment.

## Introduction

Von Willebrand disease (VWD) is the most common inherited bleeding disorder, with an estimated prevalence of 0.6 to 1.3%. 1 In patients with VWD, hemostasis is impaired due to deficiency or dysfunction of von Willebrand factor (VWF). 1 The disease shows great heterogeneity, with the severity of bleeding ranging from mild to very severe. 2 Replacement therapy with a VWF-containing concentrate is widely used to treat or prevent bleeds in people with VWD, 3 4 with long-term prophylaxis recommended in patients with a history of frequent and severe bleeds. 5

The development of neutralizing antibodies (inhibitors) against VWF is a rare but potentially serious complication of replacement therapy in VWD patients that can lead to severe anaphylactic reactions and the patient can become unresponsive to VWF-containing concentrates. 6 7 8 Data on inhibitor development and management in VWD are, however, rather limited. VWF inhibitors have been reported exclusively in patients with type 3 VWD 9 except for one case in a type 2B patient. 10 The estimated prevalence of VWF inhibitors in type 3 patients is generally in the range of 5 to 10%, although a prevalence of 19.2% was reported in one study. 6 11 12 Improving our understanding of inhibitor development remains an important goal in VWD management.

wilate, a plasma-derived VWF/factor VIII concentrate (pdVWF/FVIII) containing VWF and FVIII in a physiological 1:1 activity ratio, is licensed for on-demand treatment, surgical prophylaxis, and regular prophylaxis in patients with VWD. 13 14 The prospective, 12-month WIL-31 study (NCT04052698; WILPROPHY) evaluated the efficacy and safety of wilate prophylaxis in patients with VWD who had received prior on-demand treatment with any pdVWF/FVIII for 6 months in the prospective WIL-29 study (NCT04053699). Prophylaxis with wilate reduced the annualized bleeding rate (ABR) by 84% compared with on-demand treatment in children and adults with VWD of all types. 15 No inhibitors developed during the course of WIL-29 or WIL-31 studies. 15

Here we report the cases of two brothers who, during the WIL-31 study, were found to have pre-existing VWF inhibitors.

## Case Summaries

The brothers were managed at the same treatment center in Lebanon. Both the boys, coming from a family of a consanguineous marriage, had been diagnosed with VWD shortly after birth. Both the parents have been diagnosed with VWD, and based on the family history, the boys were suspected to have type 2A VWD. Laboratory results are not available for both the parents. The father had experienced gingival bleeding, several nosebleeds which required nasal wicks, excessive bleeding after a tooth extraction which required a blood transfusion, and gastrointestinal bleeding which led to hospitalization and treatment with a VWF concentrate and a blood transfusion. The mother had experienced rare episodes of epistaxis during childhood and adolescence which were resolved with compression, heavy menstrual bleeding, and postpartum hemorrhage in one of four childbirths which was treated with a blood transfusion. There was no history of VWF or FVIII inhibitors in the family, although testing was not performed.

Both the boys have been treated with on-demand VWF-containing concentrates since their diagnosis. Available data on historical VWF activity and antigen levels date back to 2019 and 2006, respectively. Both the patients had low VWF activity levels (VWF:RCo: 1%). The older brother (hereafter called Patient A) also had low VWF antigen levels (VWF:Ag: 10%), while VWF antigen levels were not measured in his younger brother (Patient B). At the start of the WIL-29 study, Patient A was 14 years old and weighed 38 kg, while Patient B was 11 years old and weighed 26 kg.

During the 6-month WIL-29 study, Patient A experienced seven minor spontaneous nosebleeds, of which five (71%) were treated with 32 to 63 IU/kg of a pdVWF/FVIII concentrate with a 2:4:1 ratio of VWF:FVIII (Humate P) and tranexamic acid. Patient B experienced six minor spontaneous nosebleeds during WIL-29 of which four (67%) were treated with a pdVWF/FVIII concentrate and tranexamic acid, three with 44 to 46 IU/kg Humate P, and one with 19 IU/kg wilate. The treatment efficacy was rated as “excellent” for all treated nosebleeds.

After completion of the WIL-29 study, both the boys entered WIL-31 (Patient A: 15 years old, 43 kg; Patient B: 11 years old, 29 kg) and received twice weekly prophylaxis with wilate (Patient A, 72 IU/kg per week; Patient B, 66 IU/kg per week). The measured VWF ristocetin cofactor (VWF:RCo) activity at the screening visit was 1 IU/dL for both the boys.

During prophylaxis in WIL-31, the ABRs of Patients A and B were reduced by 85 and 91%, respectively, compared with prior on-demand treatment. During the 12-month WIL-31 study, Patient A experienced one major and one minor nosebleeds, each lasting 1 day. The major nosebleed was treated with a total of 106 IU/kg wilate (two infusions, 42 and 64 IU/kg) and tranexamic acid (1,000 mg, three times for 1 day only); the minor nosebleed was treated with one infusion of 38 IU/kg wilate. Patient B experienced one minor nosebleed which lasted 2 days and was treated with one infusion of 32 IU/kg wilate and 500 mg tranexamic acid. The efficacy of treatment was rated as “excellent” for both minor nosebleeds and “moderate” for the major nosebleed.

**Fig. 1:**
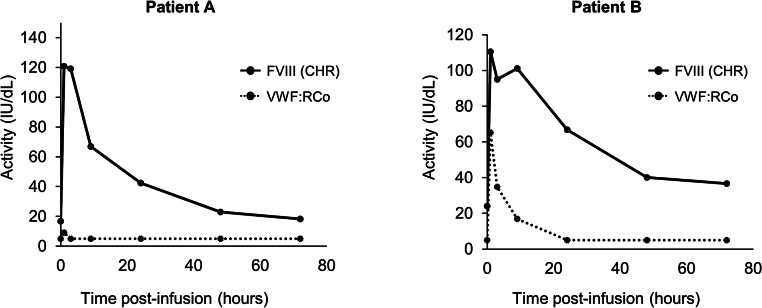
Pharmacokinetics of VWF and FVIII at month 0 in both the patients. Pharmacokinetic assessment followed a single 60 IU/kg dose of wilate. CHR, chromogenic assay; FVIII, factor VIII; IU, international units; VWF, von Willebrand factor; VWF:RCo, von Willebrand factor ristocetin cofactor activity.

Blood samples were collected at baseline (month 0) and after 1, 2, 3, 6, 9, and 12 months of prophylaxis for later pharmacokinetic (PK) *in vivo* recovery (IVR). The results of the PK analyses of the month 0 samples were received approximately 3 months after the patients had started receiving wilate prophylaxis. Pre-injection FVIII and VWF levels at the month 0 visit were low for Patient A (FVIII: 12 IU/dL; VWF:RCo: <5 IU/dL) and Patient B (FVIII: 16 IU/dL; VWF:RCo: <5 IU/dL). The results revealed virtually no increase in VWF activity in Patient A (maximum VWF:RCo activity 9 IU/dL; IVR 0.07 kg/dL), and a small rise in Patient B (maximum VWF:RCo activity 65 IU/dL; IVR 1.02 kg/dL) after administration of 60 IU/kg wilate ( Fig. 1 ). FVIII activities increased to maximal levels of 128 (IVR 2.0 kg/dL) and 110 IU/dL (IVR 1.6 kg/dL) in Patients A and B, respectively ( Fig. 1 ). The mean half-life of FVIII across all visits as measured by the chromogenic assay was 8.2 hours for Patient A, and 20.6 hours for Patient B. The expected mean half-life of FVIII with wilate is approximately 20 hours for patients with any type of VWD. 16

The month 0 PK data were discussed between the treating physician and the coordinating investigator. Given that wilate prophylaxis appeared to be efficacious and there was no clinical suspicion of inhibitors, it was decided that both the patients could continue in the study and that inhibitor testing should be performed at the next scheduled study visits. Samples from the 6-month visits tested positive for VWF inhibitors. Subsequent testing of the retention samples taken at the WIL-31 screening visits confirmed that both the patients already had VWF inhibitors at study entry. Inhibitor titers had not been measured during WIL-29, but there was no clinical suspicion of inhibitors. The investigator and coordinating investigator recommended that both the patients should remain in the study due to the continued clinical benefits of wilate prophylaxis. Inhibitor testing was repeated at the 9- and 12-month visits. Patient A remained inhibitor positive, whereas Patient B had no detectable inhibitor at the 12-month visit.

**Fig. 2:**
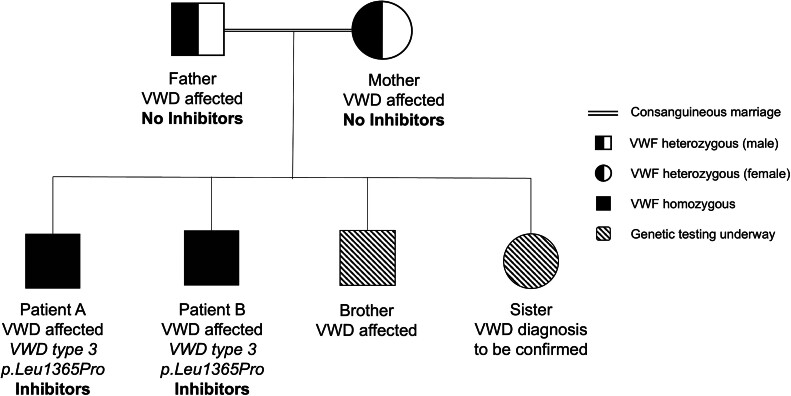
Family tree including next-generation sequencing results for the two brothers with inhibitors. VWD, von Willebrand disease.

Following the confirmation that both the brothers had VWF inhibitors, it was decided to re-assess their diagnosis of type 2A VWD. Next-generation sequencing was performed in Bonn, Germany, and the results revealed that both the brothers had a homozygous genetic variant *p.Leu1365Pro* VWF ( Fig. 2 ).

Both the patients were reclassified as type 3 VWD after the study completion visit.

## Discussion

We present here the cases of two brothers with VWD who were found to have VWF inhibitors, despite the absence of clinical symptoms, while taking part in a clinical study of wilate prophylaxis. In both the cases, inhibitor testing was prompted by unusual PK results, and the inhibitors were subsequently found to have been present prior to the start of prophylaxis. Since earlier blood samples were no longer available, it was not possible to determine the timepoint of inhibitor development in either patient. Prior to the prophylaxis study, both the boys had been treated on-demand for 6 months during the WIL-29 run-in study. During this time, Patient A had been treated five times with the pdVWF/FVIII concentrate Humate P, while Patient B had been treated three times with Humate P and once with wilate. Both the boys had also received on-demand treatment with Humate P for a number of years prior to the WIL-29 study. Despite the presence of VWF inhibitors, both the patients achieved a reduction of ≥85% in ABR during wilate prophylaxis compared with on-demand treatment.

VWF inhibitors are generally rare, with rates as low as 0.4% across all VWD types observed in a retrospective analysis of 1,650 VWD patients. 17 Inhibitors are more common in type 3 VWD, 11 with prevalences ranging from 6.2 to 19.2% in different cohort studies. 6 12 17 None of the 33 patients in the WIL-31 study developed *de novo* VWF inhibitors while on wilate prophylaxis, consistent with previous data from clinical studies with wilate. No inhibitors were reported in 97 patients across three clinical trials. 16 In a real-world study of wilate, 2 of 63 patients developed VWF inhibitors. 18 One patient, undergoing on-demand treatment, tested negative at a follow-up visit. The second patient, with type 3 VWD, tested positive on two occasions but exhibited no clinical symptoms. This patient continued wilate prophylaxis for approximately 2 years, with the treatment effectiveness being rated as “excellent” or “good.” 18

The development of VWF inhibitors may be associated with severe anaphylactic reactions to VWF-containing products. 6 7 In the two boys reported here, wilate was well tolerated, with no anaphylactic reactions or severe treatment-related adverse events, consistent with the whole WIL-31 study population. 15

VWF inhibitors may disrupt hemostatic function through two distinct mechanisms. First, inhibitors impair VWF's role in platelet adhesion and aggregation, which is crucial in primary hemostasis. Second, inhibitors reduce VWF's protection of FVIII, leading to decreased FVIII levels. 19 20 FVIII circulates in the plasma bound to VWF which shields FVIII from premature clearance and aids in the delivery of FVIII to coagulation sites. 21 22 23 Effective bleed prevention during wilate prophylaxis could be due, at least in part, to the rise in FVIII activity levels. The rise in FVIII levels may also contribute, together with the use of tranexamic acid, to the effective treatment of acute bleeds, despite low VWF activity levels.

The development of VWF inhibitors is a complex process influenced by various factors, including VWD type, duration of replacement therapy, and the specific genetic variant. 6 Several studies have documented a range of genetic variants in VWD patients with VWF inhibitors. 7 12 24 25 Typically, underlying genetic defects associated with inhibitors are null variants such as large deletions, nonsense mutations, or splice site variants, with research indicating that VWF inhibitors are more frequent in patients with partial or complete VWF gene deletions than in those with other variants. 7 The occurrence of VWF inhibitors in patients with a homozygous missense variant is rare and has only been reported in one patient previously. 12 In the 3WINTER-IPS cohort, the majority of patients with VWF inhibitors (11/14) were homozygous for a null allele, with one patient homozygous for a missense variant. 12 Contrarily, another study in type 3 patients reported no VWF inhibitors in patients with large partial gene deletions. 24 Additionally, frameshift variants have been reported in patients with VWF inhibitors. 25

Both the brothers were found to have a homozygous *p.Leu1365Pro* VWF variant. This variant has been reported in a small number of other cases, including a female patient with type 1 VWD in the US, 26 a male patient with type 3 VWD in Mexico, 27 a female patient with type 2M in Finland, 28 and a female patient with combined VWD and factor V deficiency in Germany. 29 The clinical impact of the *p.Leu1365Pro* VWF variant including its relationship to the development of VWF inhibitors remains unknown. Although the exact mechanism remains unclear, it is plausible to consider the role of additional genetic or environmental modifiers that may contribute to inhibitor development. The findings of this case report prompt further investigation into the genetic variants that predispose individuals to inhibitor development.

Interestingly, the younger patient no longer had a detectable inhibitor at the end of the WIL-31 study, suggesting the potential development of tolerance in response to repeated exposure to wilate. Although immune tolerance induction (ITI) is recommended in hemophilia A patients with FVIII inhibitors, 30 31 there is no standardized approach to ITI in VWD patients with VWF inhibitors. 32 ITI with wilate has been described previously in two brothers with type 3 VWD who developed VWF inhibitors. Both the patients received ITI with 50 IU/kg wilate daily, which resulted in a modest reduction in VWF inhibitor titers and an improvement in clinical symptoms in both the patients. 32 These findings indicate that further studies on the use of ITI in patients with VWF inhibitors may be warranted.

## Conclusion

This case report describes two brothers with VWD who were found to have pre-existing inhibitors while taking part in the WIL-31 clinical study. Despite inhibitor presence, neither patient displayed any clinical symptoms and prophylaxis with wilate led to a ≥85% reduction in ABR compared with on-demand treatment.
